# Diagnosis of *Bartonella henselae* Prosthetic Valve Endocarditis in Man, France

**DOI:** 10.3201/eid2008.130789

**Published:** 2014-08

**Authors:** Frédérique Gouriet, Pierre-Edouard Fournier, Caroline Zaratzian, Marion Sumian, Serge Cammilleri, Alberto Riberi, Jean-Paul Casalta, Gilbert Habib, Didier Raoult

**Affiliations:** Aix Marseille Université, Marseille, France (F. Gouriet, P.-E. Fournier, C. Zaratzian, J.-P. Casalta, D. Raoult);; Hôpital de La Timone, Marseille (F. Gouriet, M. Sumian, S. Cammilleri, A. Riberi, J.-P. Casalta, G. Habib)

**Keywords:** endocarditis, infectious endocarditis, ^18^fluorodeoxyglucose-positron emission tomography/computed tomography, ^18^FDG-PET/CT, Bartonella henselae, bacteria, prosthetic valve endocarditis, diagnosis

**To the Editor:** *Bartonella* spp. cause 2% of cases of blood culture–negative endocarditis (*1*). Early diagnosis of *Bartonella* spp. infectious endocarditis, is challenging, especially for patients with preexisting valvular heart disease. A diagnosis for these patients requires bacterial culture, serologic testing, or molecular detection in serum or tissue (*2*). The sensitivity and specificity of Duke modified criteria (*3*) for detecting endocardial involvement by echocardiography are not optimal, which results in decreased diagnostic accuracy (*4*).

^18^Fluorodeoxyglucose-positron emission tomography/computed tomography (^18^FDG-PET/CT), has been shown to be beneficial for diagnosis (*4*) and management of prosthetic valve endocarditis (*5*), particularly if echocardiographic findings are inconclusive (*6*). This procedure can be performed in patients of all ages by adjusting the dose of ^18^FDG to the weight of the patient. We report a case that illustrates the usefulness of ^18^FDG-PET/CT for diagnosis of *Bartonella henselae* infectious endocarditis in a patient with a prosthetic valve.

On October 18, 2012, a 56-year-old man was admitted to Timone Hospital (Marseille, France) with fatigue and weight loss (–6 kg) over the past 6 months. He had had an aortic valve replacement and a bioprosthesis was inserted in 2005 for rheumatic disease. The patient had owned a kitten for 6 months.

Laboratory findings showed moderate anemia (hemoglobin level 114 g/L), an elevated C-reactive protein level (34.5 mg/L), and polyclonal hypergammaglobulinemia. A test result was negative for rheumatoid factor. Transthoracic and transesophageal echocardiograms showed a thickened and partial aortic stenosis around the bioprosthesis.

Because infectious endocarditis was suspected, treatment with intravenous antimicrobial drugs (amoxicillin, 200 mg/kg/day for 6 weeks and gentamicin, 160 mg/day for 2 weeks) was initiated. On day 7, he was transferred to the cardiology department of Timone Hospital in Marseille, France. An endocarditis test was performed by using an endocarditis kit as described (*7*). Three routine blood cultures were negative. The patient was given a diagnosis of possible infectious endocarditis by using the Duke score (*3*).

An ^18^FDG-PET/CT scan was performed and showed ^18^FDG uptake in the aortic bioprosthesis area (Figure). Results of a whole body scan were normal. An immunofluorescence test for *Bartonella* spp. showed titers of 400 for IgG against *B*. *quintana* and *B. henselae* (*1*), and Western blot confirmed a reactivity pattern pathognomonic for *B*. *henselae* endocarditis. Results of a PCR performed with a blood sample stored in EDTA (*1*) were positive for *B*. *henselae*. On day 13, antimicrobial drug therapy was changed to oral doxycycline, 200 mg/day for 1 month and intravenous gentamicin, 160 mg/day for 15 days. On day 28, the patient was released from the hospital.

**Figure:**
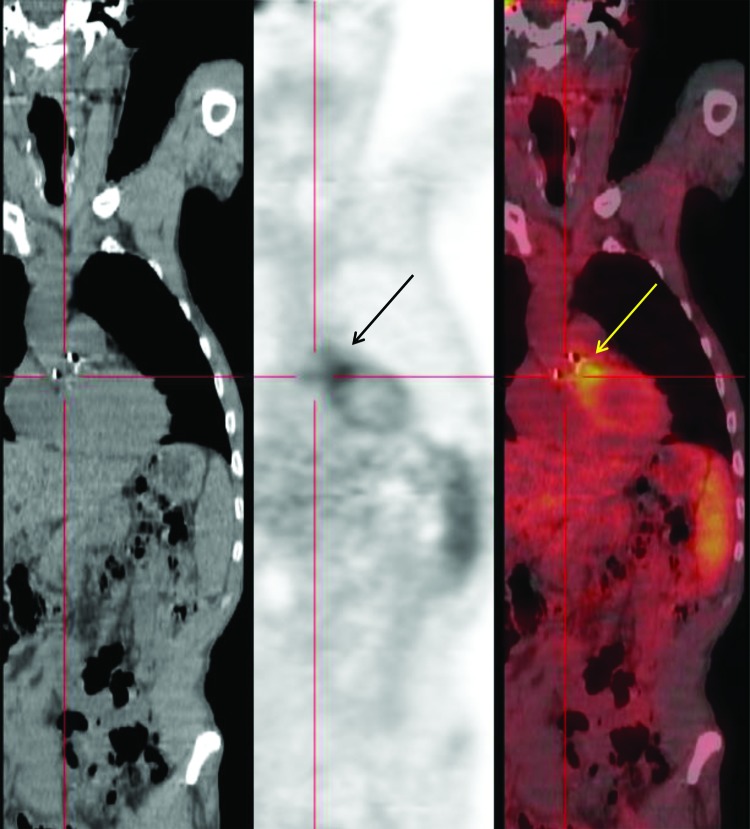
Positron emission tomography/computed tomography fusion imaging for a 56-year-old man in southern France with *Bartonella henselae* prosthetic valve endocarditis. Left panel, frontal computed tomography image showing morphologic findings. Middle panel, ^18^F-fluorodeoxyglucose–positron emission tomography (^18^FDG-PET) showing a cardiac hotspot (arrow) in relation to abnormal uptake of ^18^FDG. Right panel, fusion image combining ^18^FDG-PET and computed tomography showing localization of an aortic valve periprosthetic infection (arrow).

Two weeks later while still taking doxycycline, the patient was readmitted for a subarachnoid hemorrhage caused by a ruptured cerebral mycotic aneurysm. After arterial ligation, intravenous gentamicin was given with doxycycline for an additional 15 days. The patient recovered during this therapy.

On February 25, 2013, because of valvular stenosis, the patient underwent a new replacement with an aortic bioprosthesis. Valve culture remained sterile, but a PCR result was positive for *B*. *henselae*. Histologic analysis of the valve with Warthin-Starry stain showed no microorganisms. The patient remained asymptomatic for 2 months after surgery.

Diagnosis of possible infectious endocarditis in the patient was suspected after results of a transesophageal echocardiogram were used as a major criterion (thickened and partial aortic stenosis), and predisposing heart condition (aortic bioprosthesis) were used as a minor criterion (*3*) Because blood cultures were negative for the organism, the diagnosis of *B*. *henselae* infection was made by using serologic analysis and PCR (*7*). ^18^FDG-PET/CT was especially valuable in early diagnosis of infectious endocarditis because echocardiography showed no vegetation or abscess, a common feature of *Bartonella* spp. endocarditis in which vegetations are often small or absent. In addition, a diagnosis of infectious endocarditis remains challenging, particularly in cases of prosthetic valve infections, in which results of initial echocardiography are not useful in 30% of cases (*5*). In such cases, the diagnostic accuracy of modified Duke criteria decreases.

Use of ^18^FDG-PET/CT for diagnosis and monitoring of infectious endocarditis showed promising results, particularly for prosthetic valve infections (*5*), cardiac device–related infections (*6*), and when results of echocardiography are inconclusive or blood cultures are negative (*5*). ^18^FDG-PET/CT with abnormal uptake of FDG was proposed as a diagnostic criterion of prosthetic valve infectious endocarditis (*4*). In contrast, a negative ^18^FDG-PET/CT result does not rule out infectious endocarditis (*8*).

Use of ^18^FDG-PET/CT has been discussed mainly because of problems of sensitivity of tracer uptake in heart tissue and small vegetations (*9*). Improvements, such as patient preparation with low carbohydrate–fat diet and technical advances in the newest ^18^FDG-PET/CT scanners, may increase this sensitivity in future studies (*10*). Although ^18^FDG-PET/CT will not replace clinical evaluation, laboratory tests, and echocardiography, this procedure might be helpful in diagnosis of *Bartonella* spp. infectious endocarditis.
